# Carbohydrate Detection and Lectin Isolation from Tegumental Tissue of *Fasciola hepatica*

**Published:** 2010-03

**Authors:** A Farahnak, T Golmohamadi, MB Molaei Rad

**Affiliations:** 1 *Department of Parasitology and Mycology, School of Public Health, Tehran University of Medical Sciences, Tehran, Iran*; 2 *Department of Biochemistry, School of Medicine, Tehran University of Medical Sciences, Iran*

**Keywords:** *Fasciola hepatica*, Carbohydrate, Lectin, Pathogenesis

## Abstract

**Background:**

Fascioliasis is a chronic hepatic disease and may be resulted from mechanical/molecular parasite adhesion to host liver tissue. The aim of this study was to detect surface carbohydrate and lectin, carbohydrate-binding protein isolation that might be responsible of this molecular binding.

**Methods:**

The present experimental work was conducted in the Department of Medical Parasitology and Mycology, School of Public Health, Tehran University of Medical Sciences, Tehran, Iran. *Fasciola hepatica* parasites were collected from abattoir (Saman, Tehran, Iran) and surface mannose-carbohydrate was detected by fluorescein isothiocyanate (FITC) conjugated lectin (Lentil). Lectin of tegumental tissue from *F. hepatica* was isolated by affinity chromatography and detected by sodium dodecyl sulfate polyacrylamide gel electrophoresis (SDS-PAGE).

**Results:**

Mannose carbohydrate was observed on the surface of tegumental tissue from parasite under fluorescence microscope. Carbohydrate-binding protein or lectin with MW of 50 kDa also was isolated from homogenized tegument of helminth.

**Conclusion:**

These results are important for understanding of molecular pathogenesis of *F. hepatica* at the chronic phase of fascioliasis

## Introduction

F *asciola* (*F. hepatica* and *F. gigantica*) are the agents of human fascioliasis. The chronic phase starts when the worms reach the bile ducts. Chronic infections may result in biliary cirrhosis with scarring and fibrosis of the liver and growth deficiencies (1).

The tegument of *Fasciola* spp. is a layer of about 10 mm thick that helps the parasite to maintain its homeostasis. The surface of the tegument is highly folded and invaginated into numerous ridges and spines, which helps to increase the surface area of the tegument for the absorption and exchanging of molecules, as well as for attachment. The outer membrane covering the tegument is a trilaminate sheet about 12 nm thick, and coated with a carbohydrate-rich glycocalyx layer that also has high negative charges. The majority of antigenic proteins derived from the surface membrane and the tegument are of 97, 66, 58, 54, 47 and 14 kDa MW. While those released from the cecum are 27 and 26 kDa MW. These antigenic proteins include antioxidant enzyme, glutathione-S-transferase, fatty acid binding protein, membrane protein, muscle paramyosin protein, as well as hemoprotein and cysteine proteases (2).

As mentioned above, liver bile ducts as final habitat for *Fasciola* spp. may be seriously damaged by mechanical/molecular connection between parasite and host liver tissue. It seems that, carbohydrate-lectin (ligand proteins) interactions may have very important role in parasite adhesion to liver tissue and establishment of chronic infection of fascioliasis.

For this reason, we aimed to detect these molecules from *F. hepatica*. In the present study, fluorescence microscope was used as a detector of surface carbohydrate, and affinity chromatography method used for lectin collection from the tegument of *F. hepatica* parasites.

## Materials and Methods

### Surface carbohydrate detection of the tegument tissue of Fasciola hepatica

Live *F. hepatica* parasites were collected from naturally infected sheep livers on the day of slaughter (Saman abattoir, Tehran, Iran). Parasites were washed for a minimum of three times in phosphate buffer saline (PBS) pH 7.4, to remove host material and were stored at −80°C.

The current invitro experimental study was designed and conducted in the Department of Medical Parasitology and Mycology, School of Public Health, Tehran University of Medical Sciences, Iran in 2006-2008.

To detect of surface carbohydrate, fluorescein isothiocyanate (FITC) conjugated lectin was used. For this purpose, FITC-lentil was added to the dissected tegumental tissue of *F. hepatica* in the test tube and added in the control tube, which was containing 100 mM inhibitory sugar (mannose).The tubes were incubated at 4–8°C for 60 min and washed three times by PBS. Samples were mounted on glass slides and observed under fluorescence microscope (3).

### Lectin isolation from homogenized tegument of Fasciola hepatica

Dissected tegument of parasites was homogenized with three volumes of homogenizing buffer, PBS pH 7.4, in a glass homogenizer. The suspension were centrifuged (10000g for 30 min at 4°C) and supernatants were stored at −80°C. To provide affinity chromatography column, D-Mannose-Agarose, Sigma product number M6400 was purchased. Before use, the agarose beads were rinsed with water to remove the preservative solution, and then equilibrated with PBS. A protein solution (supernatant) was loaded onto the column in homogenizing buffer, and then washed with PBS until all non-bound protein passed through the resin (monitoring absorbance at 280 nm).

Elution of specifically bound protein was obtained by PBS containing mannose as elution buffer and detected by Coomassie blue staining of 10% polyacrylamide gel (4).

## Results

**Fig. 1:**
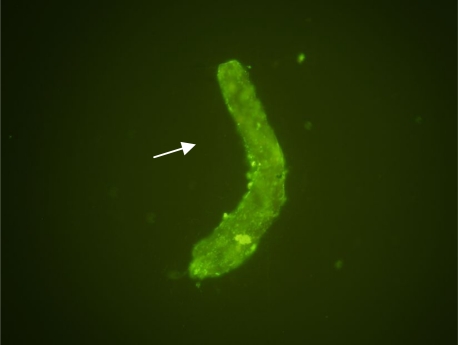
Detected mannose (Florescence foci) on the surface of isolated tegumental tissue of *F. hepatica* by conjugated lentil lectin

**Fig. 2:**
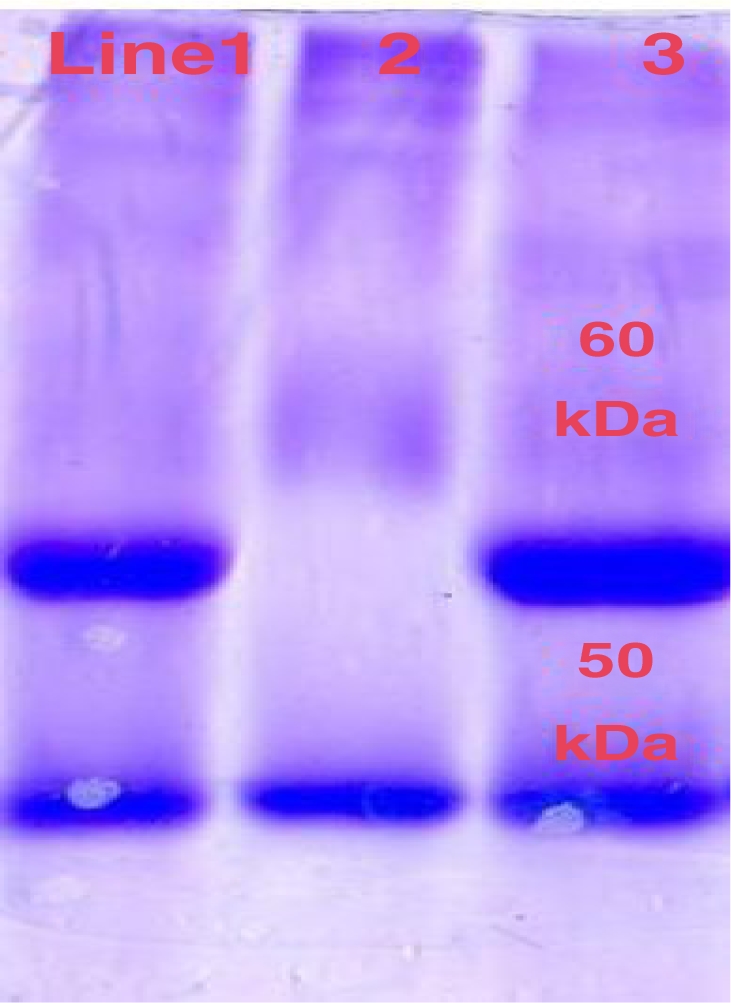
SDS-PAGE pattern of collected elution of tegumental tissue of *F. hepatica* from affinity column (Lane 2) and Sigma Protein Marker (Lane 1, 3)

Mannose monosaccharide, CH2OH (CHOH) 4CHO, was detected as surface carbohydrates at a 1/20 dilution of FITC-Lentil. The results revealed that mannose saccharid was abundant on the tegument of *F. hepatica* parasite. Mounted parasites showed high surface florescence foci however, the sample of control did not show similar feature (Fig. 1). Collected protein solution from resin showed one band protein of 50 kDa MW Using SDS-PAGE protein pattern (Fig. 2).

## Discussion

Restricted publications have been presented on the mannose monosaccharide and lectins from *F. hepatica*. Adult flukes, *F. hepatica* that were incubated in Hedon-Fleig saline containing concanavalin A (Con A) have been indicated the presence of carbohydrate complex (5). Mannose is a sugar monomer of the hexose series of carbohydrates. This sugar is present in numerous glycoconjugates including N-linked glycosylation of proteins. The mannose receptor is a calcium-dependent lectin. It has been researched as a target for vaccines (6). In the current study, existence of mannose molecules on the surface of parasite indicates its potential for molecular adhesion to host liver lectin.

Lectins are sugar-binding proteins, which are highly specific for their sugar moieties. They typically play a role in biological recognition phenomena involving cells and proteins. They may bind to a soluble carbohydrate or to a carbohydrate moiety, which is a part of a glycoprotein or glycolipid. They typically agglutinate certain animal cells and/or precipitate glycoconjugates. Concanavalin A as a lectin has been widely used as model systems to understand the molecular basis of how proteins recognize carbohydrates, because they are relatively easy to obtain and have a wide variety of sugar specificities. The many crystal structures of legume lectins have led to a detailed insight of the atomic interactions between carbohydrates and proteins (7). Detective effect, epithelium expression, adhesive specification, and anti inflammatory properties of glycoprotein have been reported from *Fasciola* spp. Enzyme-linked immunosorbent assay (ELISA) using a 27-kDa glycoprotein antigen could be a feasible diagnostic method for the early detection of bovine fasciolosis (8). Bile duct epithelium has expressed galectin-3 with different intensities, according to the different histological subtypes (9). Soluble adhesion molecules namely soluble intercellular adhesion molecule-1 (sICAM-1) and soluble E-selection (sELAM-1) have been assayed in human fascioliasis cases with or without complications (10). Results have demonstrated the potent anti-inflammatory properties of *F. hepatica* tegumental antigen and its therapeutic potential as an anti-inflammatory agent (11). In this study, D-mannose agarose, which was used for collection of lectin from parasite, was able to binding to Con A lectins from a soluble extract, therefore, the collected lectin may be Con A lectin- like protein.

In conclusion, the results of this research partially support this theory that mannose carbohydrate of *F. hepatica* may be used as receptor for specific lectin from host liver tissue and vice versa. Binding of lectin with mannose as the first molecular interaction causes parasite adhesion to liver tissue, where this process is the beginning of pathogenesis.
